# A Wearable Ultrasound Sensing System for Soft Tissue Stiffness Detection: A Feasibility Study

**DOI:** 10.3390/bios16010009

**Published:** 2025-12-22

**Authors:** Guangshuai Bao, Tongyi Xu, Xiaoyu Li, Bo Meng

**Affiliations:** Key Laboratory of Optoelectronic Devices and Systems of Ministry of Education and Guangdong Province, College of Physics and Optoelectronic Engineering, Shenzhen University, Shenzhen 518060, China; 2400233081@mails.szu.edu.cn (G.B.); tongyixu@foxmail.com (T.X.)

**Keywords:** wearable ultrasound sensing, soft tissue stiffness, ToF difference, ultrasonic indentation testing, biomechanical properties, real-time monitoring, lesion identification

## Abstract

Manual palpation serves as a conventional clinical method for assessing soft tissue stiffness; however, its results are susceptible to subjective factors and exhibit limited reliability. To achieve objective evaluation of pathological tissue stiffness, this study utilizes ultrasonic transducers to measure the time-of-flight (ToF) difference in ultrasound signals in silicone samples and ex vivo animal tissues under specific pressure gradients. A correlation model between the ToF difference and tissue stiffness was established, thereby enabling the detection of tissue stiffness. Based on this methodology, a wearable sensing system incorporating ultrasonic transducers was developed. The system applies fixed gradient pressure to human tissues via a pneumatic control unit and detects the corresponding ToF difference, allowing real-time monitoring of stiffness variations in the biceps brachii and thigh during relaxation and contraction, in the forearm during gripping and release actions, as well as in simulated lesions. This study provides a quantitative technological framework for wearable tissue stiffness monitoring, and its objective measurement characteristics offer support for clinical diagnostic decision-making.

## 1. Introduction

Wearable sensing, as an emerging technology in healthcare, has garnered significant research attention. Sensing systems developed based on diverse principles [1,2,3,4,5,6] enable continuous, distributed, and non/minimally invasive monitoring of multiple human physiological parameters [7,8], thereby supporting health status assessment and disease progression tracking. Advancing alongside sustained progress in ultrasonic transducer fabrication [9,10], circuit design [11], and algorithmic optimization [12,13], medical ultrasonography now facilitates qualitative and quantitative evaluation of core physiological parameters including anatomical structures [14], tissue properties [15], and hemodynamics [16,17]. This progress has concurrently accelerated the development of wearable ultrasonic systems [18]: J. R. Sempionatto et al. integrated ultrasonic transducers with electrochemical sensor arrays to develop an epidermal patch capable of synchronously monitoring carotid metabolites and hemodynamic parameters (e.g., blood pressure, heart rate) non-invasively [19]. The Muyang Lin team realized a fully integrated wearable ultrasound patch system (USoP) that continuously monitors central arterial pressure, heart rate, and cardiac output for up to 12 h [20]. K. W. K. Tang and colleagues developed a wearable ultrasonic device with miniaturized bioadhesive hydrogel design, achieving dimensions comparable to standard electrophysiological electrodes, which enables stable, long-term somatosensory cortical stimulation [21].

The synergistic advancement of flexible electronics, materials science, and Internet of Things (IoT) technologies is continuously expanding the capabilities of wearable devices in health monitoring. This expansion manifests in two primary dimensions: enhanced multi-parameter capture through multimodal sensing convergence and improved wearer–device compatibility via miniaturization and flexible integration. Collectively, these technological breakthroughs have laid the foundation for novel biomechanical parameter monitoring systems [22], propelling the transition toward medical monitoring characterized by heightened precision, extended operational duration, and minimized invasiveness.

Substantial evidence confirms that alterations in soft tissue stiffness are significantly associated with diverse physiological and pathological conditions, including muscle fatigue, muscular aging, inflammatory responses, and neoplastic lesions [23]. The precise quantification of soft tissue stiffness holds considerable clinical value for disease diagnosis, rehabilitation therapy, and health monitoring. However, conventional manual palpation—widely employed in clinical practice—suffers from inherent limitations such as subjective variability, low reproducibility, and inadequate sensitivity for deep-seated tissues or micro-lesions [24,25]. While existing quantitative techniques, including ultrasound elastography [26,27], magnetic resonance elastography (MRE) [28], and optical coherence elastography (OCE) [29], offer high precision, they remain constrained by reliance on bulky instrumentation and complex operational protocols. These factors impede their utility for continuous real-time physiological monitoring [30,31,32,33].

In contrast, indentation testing demonstrates distinctive advantages in the field of elasticity measurement due to its structural simplicity, operational efficiency, and capability for real-time acquisition of tissue mechanical properties [34]. Consequently, the development of a wearable soft tissue stiffness sensing system based on ultrasonic transducers addresses the limitations of current methodologies while simultaneously providing a novel solution for continuous monitoring in both clinical diagnostics and daily health management.

This study aims to design and develop a wearable soft tissue stiffness sensing system based on ultrasonic transducers. Grounded in the principles of ultrasonic indentation testing, the system leverages the efficiency of ultrasonic signal processing and the miniaturization capability of wearable devices to achieve real-time dynamic assessment of soft tissue stiffness.

## 2. Design, Optimization, and Testing of the Wearable Ultrasound Sensing System

### 2.1. Architecture and Operating Principle of the Wearable Ultrasound Sensing System

**Figure 1:**
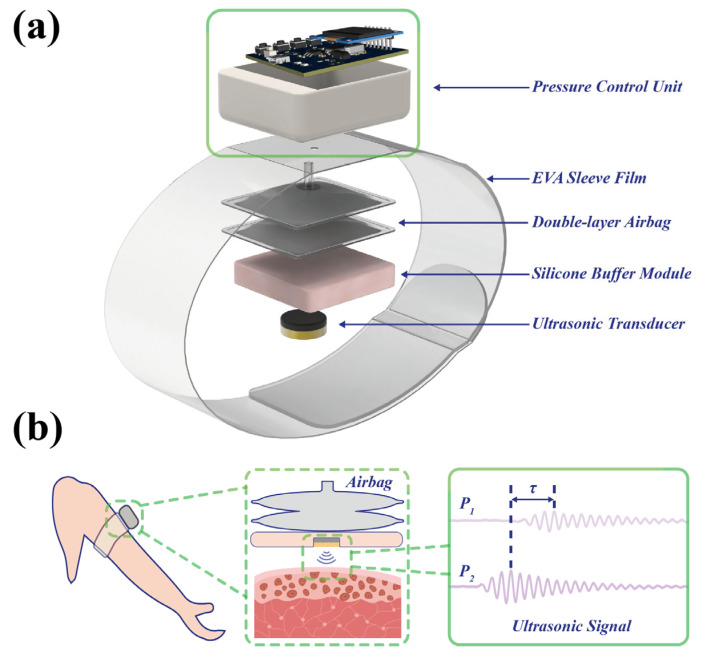
System schematic diagrams. (**a**) Wearable ultrasound sensing system schematic. (**b**) Operational principle of the ultrasonic sensing system.

The overall architecture of the wearable ultrasound sensing system is illustrated in Figure 1a, comprising five integrated components: a pneumatic control unit, an EVA film cuff, a dual-layer air chamber, a silicone buffer module, and an ultrasonic transducer. The system operates on the principle of ultrasonic indentation testing, wherein the ultrasonic transducer applies controlled pressure to target tissues while simultaneously analyzing the reflected ultrasound signals to characterize tissue elasticity. Central to this technique is the precise quantification of two fundamental physical parameters: the applied force and the resultant tissue deformation.

The classical Hertzian contact theory [35,36] provides a theoretical foundation for indentation mechanics when the following conditions are satisfied: (i) the tissue dimensions and thickness are significantly larger than the transducer’s contact area; (ii) the transducer’s elastic modulus substantially exceeds that of the tissue; (iii) the transducer maintains conformal contact with the tissue under frictionless conditions; and (iv) the tissue can be approximated as an isotropic, homogeneous material. Under these conditions, the indentation process aligns with classical elastic contact mechanics, and the relationship between the tissue’s Young’s modulus ( E ) and the mechanical input parameters—applied load ( P ) and indentation depth ( w )—can be expressed as

(1) $$E=\frac{1-\upsilon^{2}}{2a}\cdot \frac{P}{w}\;\;,$$

where w denotes the indentation depth (deformation), P represents the applied indentation force, a is the radius of the cylindrical indenter’s contact surface, v is the Poisson’s ratio of the tissue, and E is the Young’s modulus of the tissue.

However, in practical tissue stiffness measurements, several challenges arise: biological tissues typically exhibit anisotropic characteristics, ideal frictionless contact conditions between the transducer and tissue are difficult to achieve, and the Poisson’s ratio ( v ) of tissues is often challenging to determine accurately. Consequently, direct application of this formula for calculating tissue stiffness frequently fails to reflect the actual mechanical parameters reliably. To enable more robust assessment of tissue stiffness, this study introduces an alternative approach by measuring the ToF difference in ultrasonic waves under controlled pressure. A linear fitting relationship is established between tissue stiffness and the ToF difference, thereby allowing the ToF difference to serve as an accurate and practical proxy for characterizing tissue stiffness.

When an ultrasonic transducer functions as an indentation head to compress soft tissues, variations occur in the ultrasonic echoes received by the transducer. This phenomenon arises due to the reduction in thickness of the soft tissue in contact with the transducer under applied force, consequently decreasing the time required for ultrasound waves to propagate through the tissue, encounter interfaces, and return. This process can be mathematically represented as follows. Let the initial tissue thickness be denoted as h in the indentation model, and the speed of ultrasound propagation in the medium be v . After indentation, the tissue thickness becomes $h_{i}$ , with the resultant deformation defined as w in the indentation model. The corresponding time difference, termed the ToF difference τ , is illustrated schematically. Critically, τ represents the total round-trip time difference for ultrasound signals traveling to the tissue interface and returning. Thus, the following relationships hold:

(2) $$w\;=\;h-h_{i}=\frac{v\cdot \tau }{2}\;\;,$$

Within the same tissue, the ultrasonic propagation velocity ( v ) can be considered constant. Under this condition, a proportional relationship exists between the deformation ( w ) and the ToF difference ( τ ). Since this study characterizes tissue stiffness by measuring the ToF difference under a fixed pressure differential, the influence of variations in acoustic velocity among different tissue types does not need to be considered in this specific application context. In subsequent experiments, the degree of tissue deformation can be inferred by directly measuring the τ value. The operational principle of this system is illustrated in Figure 1b.

### 2.2. Measurement of Silicone Sample Stiffness Using Ultrasonic Transducers

Prior to deploying the wearable soft tissue stiffness sensing system in applied research, validation experiments on the ultrasonic indentation apparatus integrated into this system were essential. The system employs a cylindrical ultrasonic transducer with a central frequency of 2 MHz, featuring a base radius of 10 mm and a height of 8 mm. To verify the transducer’s capability for targeted soft tissue stiffness identification, stiffness measurements were performed on silicone samples. Samples with Shore hardness values spanning 0–60 HA (in increments of 10 HA) and dimensions of 48 mm × 48 mm × 10 mm were selected. To minimize ultrasonic signal attenuation in air, an acoustic couplant was applied at the interface between the transducer and the test sample surface. Each sample was positioned on a test platform, and a robotic arm controlled the vertical displacement of the ultrasonic indentation head to apply precise pressure.

**Figure 2:**
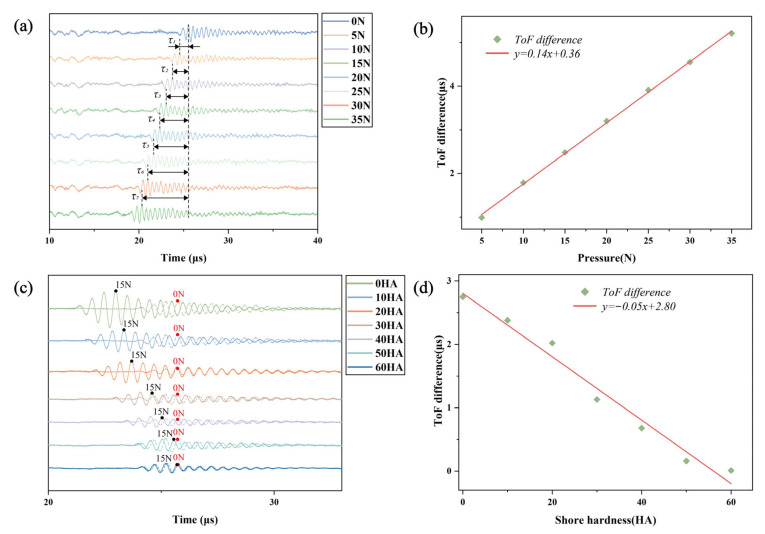
Silicone sample stiffness measurements. (**a**) Ultrasonic signals of the 10 HA silicone rubber sample under applied pressures ranging from 0 to 35 N. (**b**) The corresponding fitted curve of the ultrasonic signal ToF difference under respective pressures. (**c**) Ultrasonic signals of silicone rubber samples with different Shore hardness values (0–60 HA) at 0 N and 15 N applied pressure. (**d**) The corresponding fitted relationship between the ToF difference in the ultrasonic signals and the Shore hardness.

Initially, indentation forces ranging from 0 to 35 N (in increments of 5 N) were applied to a silicone sample with a Shore hardness of 10 HA. The corresponding ultrasonic signals are shown in Figure 2a. As the indentation force increased from 0 N to 35 N, the ToF difference ( $\tau_{i}$ ) relative to the 0 N baseline progressively increased. A linear fitting relationship between the applied pressure and the ToF difference is presented in Figure 2b. This trend reflects the physical mechanism whereby increased indentation force induces greater sample deformation, reduces thickness, and consequently extends the ToF difference. The experimental results validate the proportional relationship between the deformation ( w ) and the ToF difference ( τ ), while demonstrating the transducer’s capability to effectively distinguish different indentation forces.

Subsequently, indentation forces of 0 N and 15 N were applied to silicone samples with Shore hardness values ranging from 0 to 60 HA (in increments of 10 HA), which had been calibrated using an LX-A type Shore durometer. Ultrasonic signals under both loading conditions were recorded for each hardness level, with all signals summarized in Figure 2c. To facilitate comparison of the ToF differences, the signals were temporally aligned, where dashed lines represent the 0 N baseline and solid lines correspond to the signals under 15 N loading. Figure 2d illustrates the linear fitting relationship between Shore hardness and the ToF difference. It can be observed that under identical pressure differences, samples with higher Shore hardness exhibit smaller ToF differences, indicating that the ultrasonic indentation testing method can effectively distinguish silicone samples of different stiffness levels.

### 2.3. The Measurement of the Stiffness of Isolated Animal Tissue by Means of Ultrasonic Transducers

**Figure 3:**
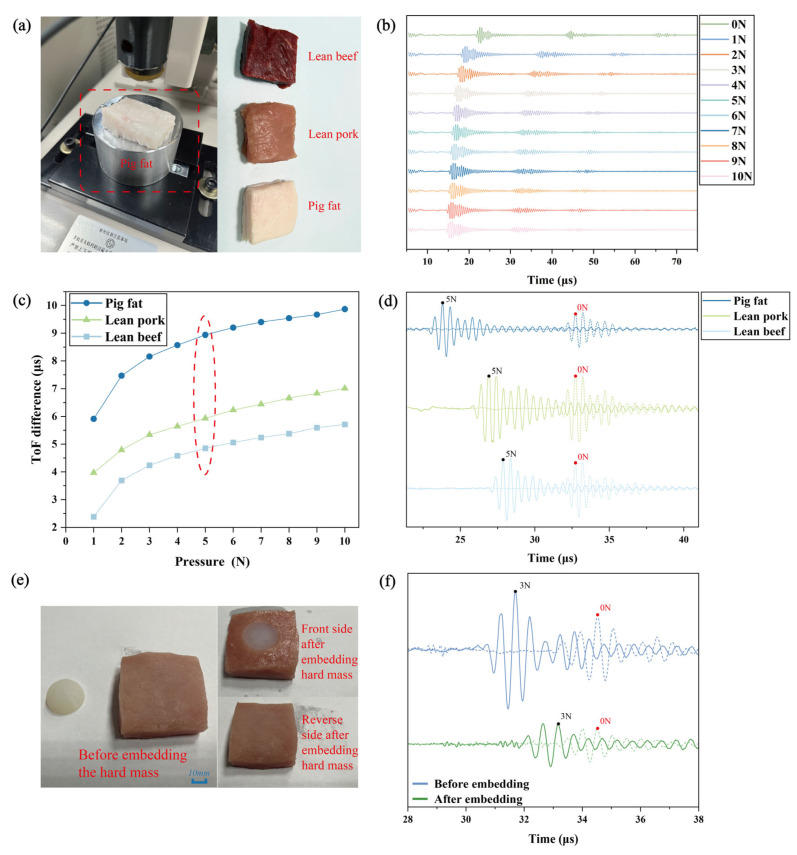
Ex vivo tissue stiffness measurements. (**a**) Photograph of isolated animal tissue specimens. (**b**) Peak phase shift in lean pork under 0–10 N pressure. (**c**) Peak phase difference across tissue types under 0–10 N pressure. (**d**) Ultrasound signals of tissue specimens at 5 N. (**e**) Simulated lesions in lean pork via embedded silicone blocks. (**f**) Ultrasound signal comparison before and after lesion simulation at 3 N.

Prior to conducting the overall system design, we continued applying the Ultrasound Indentation Method to ex vivo animal tissues. Compared to the polymer specimens, these animal tissues better mimic human tissue characteristics. Three types of animal tissue exhibiting significant stiffness differences were selected: porcine adipose tissue, porcine lean muscle, and bovine lean muscle. These tissues were trimmed into uniform 5×5×3 cm cuboids. Testing was performed using the same experimental platform as employed in the silicone sample experiments, as depicted in Figure 3a.

Ultrasonic indentation testing was initiated on each ex vivo animal tissue specimen from an initial load of 0 N. The indentation force was incrementally increased in discrete steps of 1 N until reaching 10 N, with the ultrasonic signal recorded at each discrete pressure increment. Representative ultrasonic signals corresponding to eleven discrete loading increments applied to porcine lean muscle are presented in Figure 3b; analogous signal patterns were observed for bovine lean muscle and porcine adipose tissue. As illustrated in Figure 3b, progressive signal shift occurred in the ultrasonic waveforms from porcine lean muscle with increasing indentation force. Concurrently, the magnitude of the phase shift progressively decreased. This observation aligns with findings previously obtained from silicone specimens.

The ToF differences for each ex vivo tissue relative to the 0 N baseline, measured across the applied pressure range, are plotted in Figure 3c. This graph reveals that the ToF difference increases nonlinearly with applied pressure for all tissue types, with a progressively diminishing rate of increase. This nonlinear variation mechanism can be explained as follows: for the same ex vivo tissue, an increase in applied pressure initially induces greater tissue deformation, resulting in a corresponding elevation of the ToF difference. However, as pressure further escalates, the tissue’s compressive resistance intensifies, leading to a progressive reduction in deformation magnitude under equivalent pressure increments. Consequently, the rate of ToF increase diminishes, ultimately exhibiting characteristic nonlinear behavior. To evaluate the capability of the ultrasonic transducer to differentiate between tissue types, ultrasonic signals acquired at specific forces of 0 N and 5 N are presented in Figure 3d, with temporal alignment applied for enhanced comparison. Figure 3d demonstrates a distinct hierarchy: the ToF difference for bovine lean muscle is less than that of porcine lean muscle, which in turn is less than that of porcine adipose tissue. Consequently, the tissue stiffness follows the order: bovine lean muscle > porcine lean muscle > porcine adipose tissue. This measured stiffness ranking corresponds directly to the intrinsic stiffness differences observed among the selected ex vivo tissues.

These results demonstrate that the ultrasonic indentation testing methodology effectively distinguished the relative mechanical stiffness properties of the different animal tissues under investigation.

In soft tissue pathologies, many lesions originate within the tissue interior (e.g., subcutaneous edema, lipomas, lymphadenopathy). These abnormalities are often imperceptible by visual inspection of the tissue surface but are typically accompanied by alterations in local mechanical stiffness. Consequently, this study employs tissue stiffness variation as a metric for detecting internal soft tissue lesions. Preliminary validation was conducted using ex vivo animal tissues, employing a methodological approach wherein rubber inserts were embedded within porcine lean muscle specimens to simulate internal lesions, as schematically presented in Figure 3e.

Porcine lean muscle tissue was sectioned into dimensions consistent with previous ex vivo experiments (5 × 5 × 3 cm cuboids). Baseline measurements were first performed on non-embedded specimens, recording ultrasonic signals at initial 0 N and under a 3 N indentation force. Subsequently, a simulated lesion model was constructed using a hemispherical silicone specimen (Shore hardness: 30 HA). Precise hemispherical excision matching the implant volume was performed within the tissue, followed by embedding the silicone specimen. This configuration enabled direct ultrasonic indentation testing over the embedded region by the transducer probe, with results shown in Figure 3f.

A significantly reduced ToF difference was observed post-embedding compared to the non-embedded condition, as demonstrated in Figure 3f. This indicates diminished deformation under identical indentation forces following silicone implantation. The methodology successfully differentiated between non-embedded tissue and tissue containing the embedded synthetic lesion, confirming its efficacy for detecting localized stiffness variations associated with internal pathologies.

### 2.4. Design of the Wearable Soft Tissue Stiffness Sensing System

Following the functional validation of the ultrasonic transducer, this section details the design of four core modules in the wearable system: the pneumatic control unit, EVA film cuff, dual-layer air chamber, and silicone buffer module.

**Figure 4:**
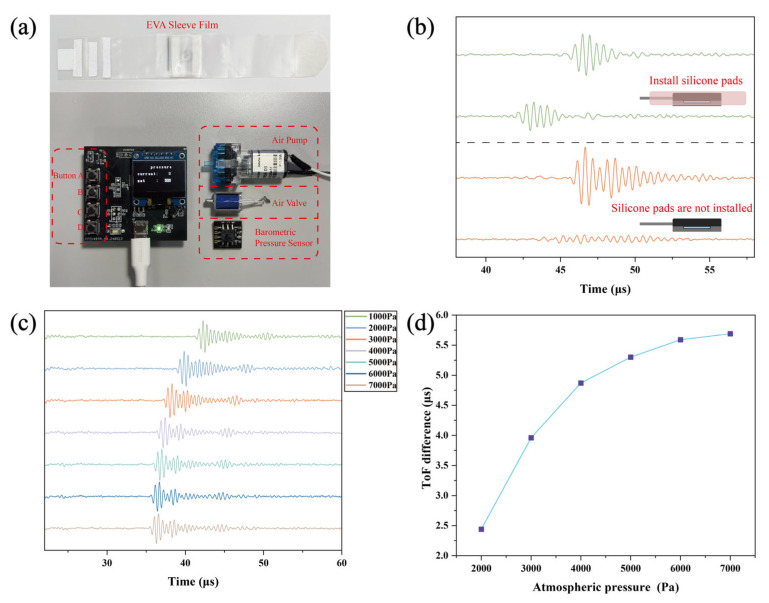
Wearable system validation. (**a**) Pneumatic control unit and cuff assembly. (**b**) Effect of silicone buffer module on ultrasound signal stability under pressure. (**c**) Forearm ultrasound signals under 1–7 kPa pressure. (**d**) ToF difference variation versus pressure.

The EVA film cuff, fabricated from 0.3 mm-thick ethylene–vinyl acetate copolymer, serves dual functions: (i) securing the sensor assembly to the human body and (ii) synergizing with the air chamber to provide a stabilized pressure environment for the ultrasonic transducer. The pneumatic control unit (Figure 4a), developed around an STM32F103C8T6 microcontroller, integrates a miniature pneumatic pump, a high-response solenoid exhaust valve, a pressure sensor, and an OLED display for real-time pressure monitoring.

To ensure stable and uniform indentation force application to the ultrasonic transducer, a dual-layer thermoplastic polyurethane (TPU) air chamber structure was implemented (Size: 50 mm × 60 mm), coupled with a silicone buffer module of Shore hardness 0 HA. Figure 4b contrasts ultrasonic signals with and without the silicone buffer module. Direct transducer loading without the buffer induced significant signal divergence and degraded waveform integrity. Conversely, the buffered configuration maintained signal clarity under all pressure conditions while enabling unambiguous peak-phase shift detection, confirming enhanced acoustic coupling efficiency.

The finalized system underwent performance testing on human forearm tissue. Under controlled pressure escalation from 1 kPa to 7 kPa (Figure 4c), the ultrasonic signals exhibited progressive leftward shifts in time-domain profiles, indicating pressure-dependent reductions in ToF difference. To quantify stiffness sensitivity, the rate of ToF difference change relative to the 1 kPa baseline was calculated for pressures spanning 2–7 kPa (Figure 4d). Within the 2–5 kPa pressure range, the ToF difference exhibited a significantly accelerated rate of change, demonstrating the system’s high responsiveness to pressure variations in this regime and its capability to effectively capture subtle differences in soft tissue stiffness. Beyond 5 kPa, however, the ToF difference trend plateaued markedly, indicating reduced sensitivity to pressure changes in higher-pressure regimes. Following holistic consideration of both the discriminative significance of ToF difference values and subject comfort thresholds, 5 kPa was identified as the optimal operational pressure.

## 3. Application Study of the Wearable Soft Tissue Stiffness Sensing System

Building upon prior research, the wearable soft tissue stiffness sensing system has been comprehensively designed and optimized across multiple dimensions, including hardware architecture, human-body compatibility, and data stability. Performance validation confirmed the system’s superior adaptability to human physiology, demonstrating its capability to accurately detect deformation in soft tissues under increasing indentation forces. This foundation supports the current application study, which focuses on validating the system’s efficacy in real-world tissue stiffness identification scenarios.

### 3.1. Stiffness Discrimination in Biceps Brachii: Extended vs. Flexed Arm States

The primary objective of this study was to develop a system for precise identification and assessment of soft tissue stiffness. The biceps brachii, a clinically significant site for lipomas, subcutaneous cysts, and physical injuries, was selected due to its diagnostic relevance in health monitoring. Accurate stiffness quantification in this region may facilitate early detection and diagnosis of pathologies

**Figure 5:**
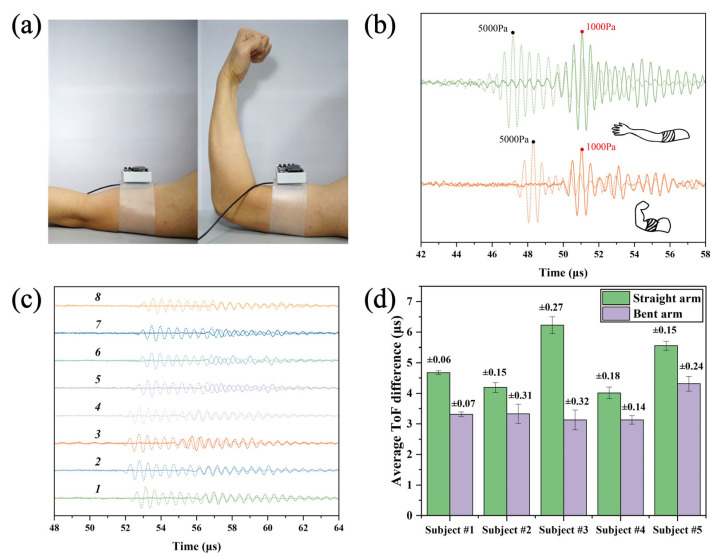
Biceps brachii stiffness discrimination. (**a**) System positioning during arm flexion and extension. (**b**) ToF difference comparison: relaxed vs. flexed states. (**c**) Ultrasonic signals obtained from Subject 4 through eight repeated measurements under arm extension conditions. (**d**) ToF difference comparison across participants during flexion/extension.

The system was secured to the biceps brachii region of participants. For baseline measurements, participants extended their arms naturally on a test platform, ensuring complete muscular relaxation (Figure 5a, left). Pressure was incrementally applied from an initial state of 1 kPa to 5 kPa, with ultrasonic signals recorded at each stage to capture stiffness data in the relaxed state. Subsequently, participants flexed their arms to induce muscle contraction, significantly increasing biceps stiffness compared to the relaxed state (Figure 5a, right). Identical pressure protocols were repeated to acquire stiffness data in the flexed state.

Comparative analysis of ultrasonic signals revealed distinct stiffness signatures between states (Figure 5b). Signals were temporally aligned to a common phase reference for clarity. The ToF difference was 3.9 μs in the relaxed state but decreased to 2.76 μs in the flexed state. This reduction in ToF difference under identical pressure differentials indicates smaller tissue deformation and thus higher stiffness during flexion—a finding consistent with biomechanical principles.

To ensure the statistical robustness of the measurements, eight repeated measurements were performed for each arm state (extended/flexed) per participant. Figure 5c displays the ultrasonic signals from one participant during arm extension, demonstrating consistent time-of-flight (ToF) differences across repeated measurements. To validate system generalizability, five healthy participants (four males aged 20–28 with BMI 23.1–32.9 and one female of comparable age with BMI 23.3) were recruited. All participants wore the wearable ultrasound system on the biceps brachii, with measurement sequence randomized between relaxed and contracted states. The grouped bar chart in Figure 5d summarizes the ToF difference results for all participants. The standard error of ToF differences across eight repeated measurements ranged from 0.06 to 0.32 μs, confirming the system’s effectiveness in distinguishing stiffness variations between arm flexion and extension states across different individuals.

### 3.2. Assessment of Forearm and Thigh Stiffness and Identification of Simulated Pathological Tissues in Arm Models

**Figure 6:**
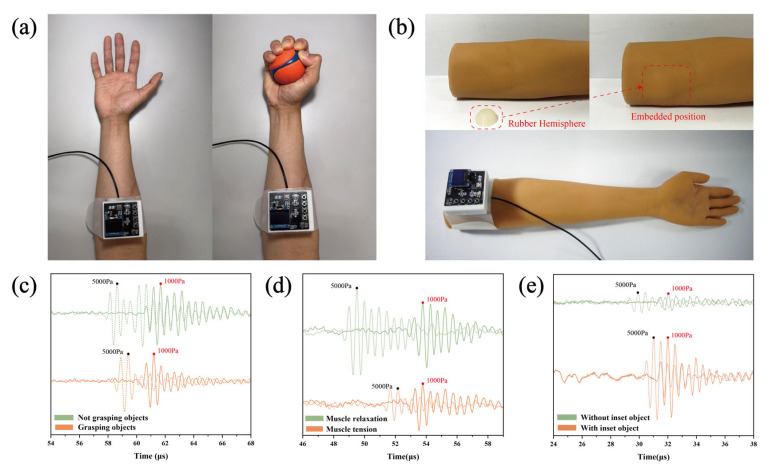
Assessment of forearm and thigh stiffness and detection of simulated lesions. (**a**) Posture comparison: relaxed palm vs. gripping. (**b**) Arm phantom with embedded rubber hemisphere (30HA). (**c**) Ultrasonic signals: relaxed arm state versus gripping state. (**d**) Ultrasonic signals: relaxed thigh state versus contracted thigh state. (**e**) ToF difference comparison: baseline vs. embedded hemisphere.

To evaluate the applicability of the wearable ultrasound sensing system for stiffness detection across diverse anatomical sites, forearm stiffness was measured during both gripping and relaxed states. As illustrated in Figure 6a, the system was secured to the forearm using the same methodology employed for biceps brachii stiffness assessment. Ultrasonic signals were recorded under 1 kPa (baseline) and 5 kPa applied pressures for both gripping and relaxed states (Figure 6c). Comparative analysis revealed a ToF difference of 3.04 μs in the relaxed state versus 1.80 μs during gripping under identical pressure conditions. The significantly larger ToF difference in the relaxed state indicates lower tissue stiffness, confirming the system’s capability to discriminate stiffness variations between gripping and relaxation phases. The system was deployed on the thigh region following the same operational protocol utilized for forearm measurements. Ultrasonic signals were recorded from the thigh muscles during both relaxed and contracted states, as illustrated in Figure 6d. The corresponding time-of-flight (ToF) differences were measured at 4.28 μs and 1.59 μs for the relaxed and contracted states, respectively. These results demonstrate the system’s capability to detect stiffness variations in thigh tissue, thereby validating its applicability for muscular assessment in the lower extremities.

Building upon prior ex vivo experiments using animal tissues embedded with silicone blocks to simulate internal pathologies, this study advanced the model by embedding rubber hemispheres (Shore hardness: 30 HA) into synthetic human arm phantoms to mimic soft tissue lesions (Figure 6b). The system was positioned above the embedded hemisphere region, and pressures of 1 kPa (baseline) and 5 kPa were applied sequentially. Ultrasonic signals acquired before and after hemisphere implantation are shown in Figure 6e. Under identical loading conditions, the ToF difference decreased from 2.11 μs pre-implantation to 1.02 μs post-implantation. This reduction in the ToF difference indicates an increase in local stiffness, which is consistent with the intended design effect of the hemisphere implantation. Although differences exist between this simulation and genuine clinical tissue pathologies, the pathological hardening characteristics observed align with those present in human soft tissue lesions [37,38], thereby further validating the effectiveness of the proposed system in detecting simulated pathological tissues via the ToF difference.

## 4. Conclusions

A wearable sensing system based on the detection of ultrasonic ToF difference was developed in this study to achieve objective assessment of soft tissue stiffness. The system enables the identification of stiffness variations in different tissues through the ToF difference under controlled pressure conditions, which has been validated by measurements on the human biceps brachii, forearm, and thigh. Furthermore, experiments simulating soft tissue lesions in arm models demonstrate the system’s potential to recognize pathological abnormalities in tissue stiffness. In contrast to conventional manual palpation, this research provides a quantitative reference method for the non-invasive clinical monitoring of soft tissue conditions.

The current system still exhibits certain limitations; future research will focus on optimizing the device structure toward miniaturization and wireless operation, and will explore the integration of machine learning methods for automated signal analysis. In addition, further validation involving real pathological tissues is required to verify its clinical applicability, while extending its detection capability to a broader range of human tissues will enhance the practical utility of the system.

## Data Availability

The experimental data is contained within the article.
